# The venous sympathetic block in chronic pain practice: absence of evidence, presence of use?

**DOI:** 10.1016/j.bjane.2025.844666

**Published:** 2025-07-30

**Authors:** Hugo Muscelli Alecrim

**Affiliations:** a Serviço de Anestesiologia Integrada (SANI), Brasília, DF, Brazil; b Active Member of the Brazilian Society of Anesthesiology (SBA), Brazil; c Active Member of the Anesthesiology Society of the Federal District (SADIF), Brasília, DF, Brazil

Dear Editor,

In Brazil, the practice commonly referred to as “Venous Sympathetic Block” (BSV in Portuguese) has gained recognition among anesthesiologists, pain specialists, and patients with chronic pain. This technique involves the slow systemic intravenous infusion of a pharmacological mixture and is entirely distinct from the regional venous sympathetic block described in existing literature. The typical combination administered includes variable doses of ketamine (0.2–1 mg.kg^-1^), dexmedetomidine (0.5–1 mcg.kg^-1^) or clonidine, lidocaine (1–3 mg.kg^-1^), and magnesium sulfate (1–3 g). Importantly, this systemic intravenous method and its precise pharmacological composition lack a standardized definition in indexed scientific literature, leading to significant variability based on practitioner preferences.

Although the relationship between ketamine and the autonomic nervous system has been the focus of studies with counterintuitive findings, it is appropriate to begin with a note of perplexity: the notion of suppressing sympathetic activity using a pharmacological agent classically classified as sympathomimetic.^1^, ^2^, ^3^ Perhaps this is merely a semantic diversion ‒ a lateral digression that, while intellectually valid, lies outside the central scope of this text: how is the routinely employed BSV described and supported in the scientific literature?

On May 23, 2025, a search on PubMed for the term “*bloqueio simpático venoso*” (Portuguese) yielded no results.

On the same date, a search for the English term “venous sympathetic block” returned 275 publications. However, a title-by-title review revealed that none of them pertained to the clinical practice in question. The alternative term “sympathetic venous block” produced 438 results, which were likewise irrelevant to the subject of this analysis.

A Google search in Portuguese finally led to a case report published in the Annals of the Scientific Week of the *Faculdade de Medicina de Campos* (2024), titled “Venous sympathetic block in the treatment of chronic pain in a patient with fibromyalgia: a case report”. The brief text defines venous sympathetic block as “slow infusion of drugs such as lidocaine, ketamine, and clonidine via the venous route. This procedure promotes, in addition to sympathetic blockade, anesthesia of vascular endothelial nerve endings, analgesia, and vasodilation, leading to pain reduction or elimination” (free translation from Portuguese into English). Unfortunately, the document does not include any references.^4^

Considering that the above case report dates from 2024 and that BSV has been performed for several years, it is not possible to infer that current clinical practice originates from or is based on this document.

In keeping with our zeitgeist, a search was conducted using the Investigate feature of ChatGPT Pro and identified publications relevant to regional venous sympathetic block, as well as the same case report previously mentioned.^4^

In the widely used Unified Supplementary Health Terminology Table (TUSS), we find the entry “Sympathetic block via venous route” (code 31602177).^5^ However, this item is listed under a subgroup of surgical and invasive procedures, which suggests it refers to regional venous sympathetic block. Yet once again, there is no bibliography that clearly defines the concept underlying the code, nor is there any explanatory text indicating to which therapies the code does or does not apply.

In light of the absence of relevant entries in indexed databases and the limitations of this brief exploratory investigation, we encourage academic centers to investigate the physiological rationale, safety profile, and effectiveness of this practice through clinical trials and pharmacological analyses.

## Conflicts of interest

The authors declare no conflicts of interest.
